# Evolution and disappearance of sympatric *Coregonus albula* in a changing environment—A case study of the only remaining population pair in Sweden

**DOI:** 10.1002/ece3.5745

**Published:** 2019-10-29

**Authors:** Bo Delling, Stefan Palm

**Affiliations:** ^1^ Department of Zoology Swedish Museum of Natural History Stockholm Sweden; ^2^ Swedish University of Agricultural Sciences Department of Aquatic Resources Institute of Freshwater Research Drottningholm Sweden

**Keywords:** climate, *Coregonus*, microevolution, microsatellites, morphology

## Abstract

During the past 50 years, Fennoscandian populations of spring‐spawning Baltic cisco (*Coregonus albula*), sympatric to common autumn‐spawners, have declined or disappeared; for example, three out of four known spring‐spawning populations in Sweden are regarded as extinct. Over the same period, the climate has changed and populations have been subject to other anthropogenic stressors. We compared historic (1960s) and recent (1990–2000s) morphological data from the still‐existent sympatric cisco populations in Lake Fegen, Sweden. Phenotypic changes were found for spring‐spawners making them more similar to the sympatric autumn‐spawners that had remained virtually unchanged. Based on results for other salmoniform fishes, a phenotypically plastic response to increased temperature during early development appears unlikely. The recent material was also analyzed with microsatellite markers; long‐term effective population size in spring‐spawners was estimated to be about 20 times lower than autumn‐spawners, with signs of long‐term gene flow in both directions and a recent genetic bottleneck in spring‐spawners. We suggest the change toward a less distinct phenotype in spring‐spawners to reflect a recent increase in gene flow from autumn‐spawners. Time since divergence was estimated to only *c*. 1,900 years (95% CI: 400–5,900), but still the Fegen populations represent the most morphologically and genetically distinct sympatric populations studied. Consequently, we hypothesize that less distinct population pairs can be even younger and that spring‐spawning may have repeatedly evolved and disappeared in several lakes since the end of the last glaciation, concurrent with changed environmental conditions.

## Graphical abstract

We compared historic (1960s) and recent (1990–2000s) morphological data from the still‐existent sympatric cisco populations in Lake Fegen, Sweden. Phenotypic changes were found for spring‐spawners making them more similar to the sympatric autumn‐spawners (that had remained virtually unchanged). The recent material was also analyzed with microsatellite markers. There were signs of ongoing gene flow in both directions, and the change toward a less distinct phenotype in spring‐spawners likely reflects increased gene flow from autumn‐spawners.

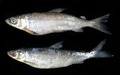

## 1 INTRODUCTION

Vast areas of the northern hemisphere, including the Fennoscandian Peninsula and the Baltic basin, were covered by glacier ice until about 18,000 yBP when the ice started to recede (Storch, Omstedt, Pawlak, & Reckermann, 2015). Deglaciation allowed for the reestablishment of flora and fauna. Subsequently, this region has exhibited considerable climate variation with both warmer and colder periods (Mauri, Davis, Collins, & Kaplan, 2015) affecting faunal and floral composition. More recent temperature variation (about ± 0.5°C) includes the “Medieval Warm Period” (900–1350 AD) and the “Little Ice Age” (1550–1850 AD) (Niedźwiedź et al., 2015). Within a contemporary timeframe, the increase in temperatures recorded since the mid‐20th century (IPPC, 2014) marks the beginning of a gradually warmer period.

Numerous studies have dealt with anthropogenic driven climate change and its recorded or expected consequences on biodiversity, such as range expansions and extinction of species or local populations (McLean, Lawson, Leech, & Pol, 2016; Pecl et al., 2017). In addition, anthropogenic influences on the environment have been shown to induce phenotypic changes at higher rates compared with what is expected due to “natural” processes (Hendry, Farruiga, & Kinnison, 2008).

Salmoniform fishes in general and coregonids in particular are renowned for their phenotypical diversity in the rather species‐poor and cold water environments they inhabit. For example, several “kinds” of chars, whitefishes and ciscoes are recognized within the *Salvelinus alpinus*, *Coregonus lavaretus*, and *Coregonus albula* complexes, respectively (Jonsson & Jonsson, 2001; Kottelat & Freyhof, 2007; Svärdson, 1998). Within these complexes sympatric forms are commonly present, segregated with respect to genetic markers, feeding habits, spawning time/‐depth and morphological characters related to ecological adaptation. These sympatric forms have either been classified as distinct endemic species (Hudson, Vonlanthen, & Seehausen, 2011), distinct but allopatric widespread species (Svärdson, 1998) or plasticity within a single species (Østbye, Bernatchez, Næsje, Himberg, & Hindar, 2005). Irrespective of classification, genetic data largely speak in favor of independent recent postglacial evolution of sympatric populations in different lakes, that is, a particular morph is more closely related to other sympatric morphs than to similar morphs in other lakes (Gíslason, Ferguson, Skúlason, & Snorrason, 1999; Østbye et al., 2006; Vuorinen, Himberg, & Lankinen, 1981).

Within the *C. albula* complex (ciscoes, vendaces), the most common and well‐studied case of sympatric forms is related to differences in spawning period. In these cases, a common autumn‐spawner typically coexists with a spring‐ or winter‐spawning population (Schulz et al., 2006; Svärdson, 1979; Vuorinen et al., 1981). Ciscoes with deviating spawning time are known from Germany (Schulz et al., 2006), Sweden (Svärdson, 1979), Norway (Huitfeldt‐Kaas, 1927), Finland (Vuorinen et al., 1981), and Russia (Airaksinen, 1968) (Table A1). Some spring‐spawning forms have also been described as distinct species (Schulz & Freyhof, 2003; Svärdson, 1979; Thienemann, 1933). Among the most well‐studied cases in Germany, Finland, and Sweden, genetic data indicate postglacial independent origins of sympatric spring‐ and autumn‐spawning populations (Delling, Palm, Palkopoulou, & Prestegaard, 2014; Schulz et al., 2006; Vuorinen et al., 1981). However, little is presently known on how and when these coexisting populations have evolved, if they are completely reproductively isolated, and their degree of resilience to environmental perturbations.

So far, the most detailed studies of sympatric ciscoes have been carried out in Lake Stechlin, Germany; in addition to physiological adaptations (Ohlberger, Mehner, Staaks, & Hölker, 2008; Ohlberger, Staaks, Petzoldt, Mehner, & Hölker, 2008), only slight morphological differences between these coexisting cisco forms could be determined, tentatively representing adaptations to different microhabitats (Helland, Vøllestad, Freyhof, & Mehner, 2009). Hence, besides marked differences in spawning time, it is intriguing that sympatric cisco populations lack the more apparent ecological niche separations typically seen in coexisting whitefishes and chars (e.g., piscivorous, benthivorous, and planktivorous morphs) often manifested by striking differences in body size and morphology related to the feeding apparatus (Markevich, Esin, & Anisimova, 2018; Snorrason et al., 1994; Svärdson, 1979).

Delling et al. (2014) studied Baltic cisco populations, including the four known Swedish cases with sympatric populations. The spring‐spawning form is extinct in three of these lakes, but archived scale samples made analyses of mtDNA variation possible. Both spring‐ and autumn‐spawners from all four lakes and additional lakes (with only autumn‐spawners) in south‐central Sweden were fixed for a few closely related haplotypes not found elsewhere in Sweden. Furthermore, these haplotypes seem more closely related to those in North American *Coregonus sardinella*. Contemporary microsatellite data for Swedish populations were also congruent with the distribution of mtDNA haplotypes, showing a striking dichotomy between samples from south‐central (higher altitude) lakes compared with other parts of Sweden, regardless of spawning time (Delling et al., 2014).

**Figure 1:**
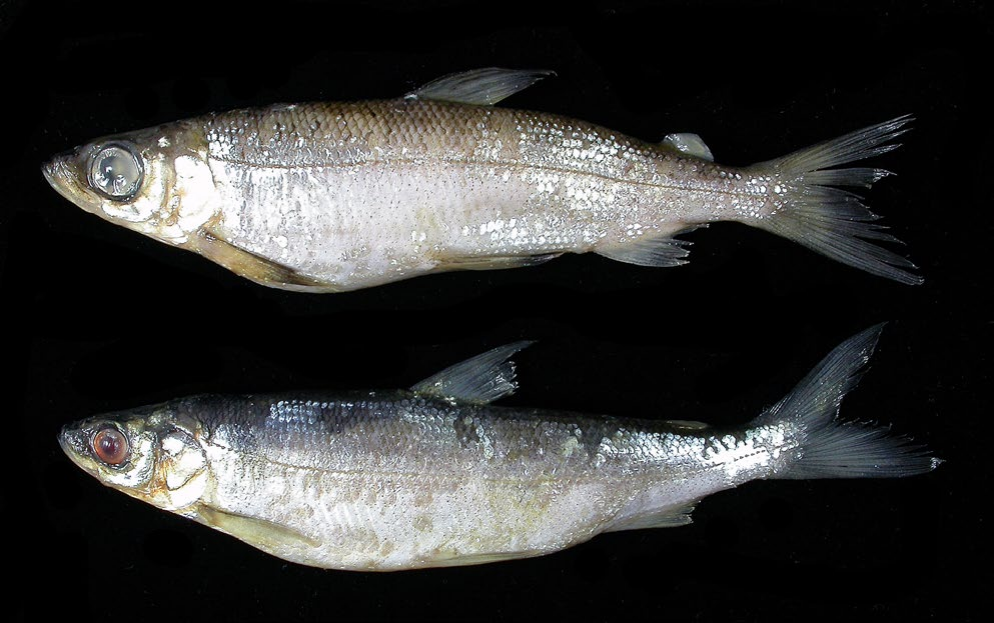
Sympatric spring‐spawning (top) NRM 53995 (127 mm *Sl*) and autumn‐spawning (bottom) NRM 54000 (131 mm *Sl*) Baltic cisco, *C. albula* from L. Fegen. The spring‐spawning population is characterized by (on average) larger eyes and larger heads

**Figure 2:**
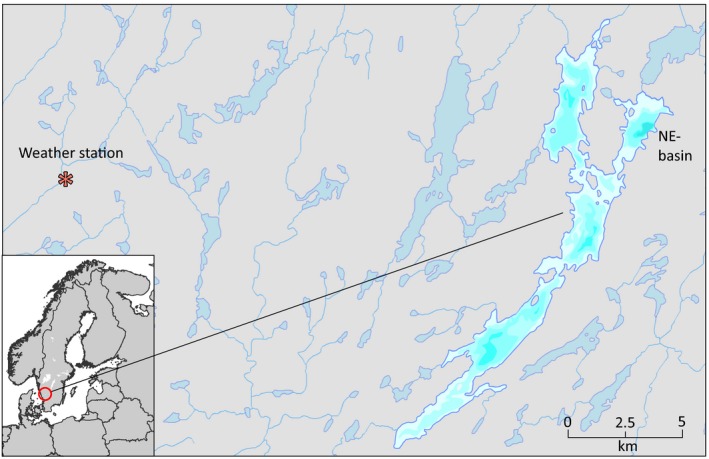
Lake Fegen (23.5 km^2^, 132 m above sea level, max depth 36–38 m) with surrounding waters in southern Sweden. Analyzed air temperatures were taken from the nearest weather station “SMHI Fagered” (marked with an asterisk). Spring‐spawning *C. albula* are mainly found in the deepest NE basin, whereas autumn‐spawners exist in all parts of the lake

The single extant population of spring‐spawning cisco in Sweden (Figure 1) inhabits Lake Fegen (Figure 2). Together with the extinct populations from Lakes Ören, Stora Hålsjön, and Åsunden, it was formally described as *Coregonus trybomi* by Svärdson (1979). The species description was mainly based on spawning time, but it was noted that the Fegen spring‐spawners exhibited a particularly distinct morphology. Most striking was a proportionally larger eye compared with other spring‐spawners and the common autumn‐spawning form in Fegen and other lakes (Figure 1). A larger eye has also been reported for one winter‐spawning population in Finland (Airaksinen, 1968), whereas the spring‐spawning populations in Ören and Åsunden (no data exist from St Hålsjön) showed no difference in eye size compared with their sympatric autumn‐spawners (Svärdson, 1979). Differences in vertebral counts have also been described, again most strongly expressed in Fegen, with spring‐spawners having on average 3.3 fewer vertebrae compared with the sympatric autumn‐spawners (Svärdson, 1979).

The extinction of spring‐spawners in Ören, Åsunden, and St Hålsjön in recent decades (1960–1990) have been explained by extrinsic factors such as pollutants, eutrophication, and introduction of non‐native fish species (Fjälling, 1988; Svärdson, 1979). The present status of winter‐ and spring‐spawning populations in Finland is largely unknown, but there are indications that they are declining or have disappeared (M. Himberg, Åbo Akademi, personal communication).

The rapid disappearance of three out of four known spring‐spawning populations in Sweden and the similar situation in Finland has raised concerns and questions regarding the status of the still surviving sympatric population pair in Lake Fegen. Also this lake has been subject to anthropogenic impacts, for example, in terms of repeated introductions of predatory pikeperch, *Sander lucioperca*, since the 1940s, and eutrophication (Thörne & Carlsson, 2004). Survey gill net fishing in 2014 suggested a decline in the overall abundance of *C. albula* compared with 2003, and based on morphologic data obtained from a subsample (mainly from the NE basin), only some 2%–4% of the 2014 catch were classified as spring‐spawners (B. Delling, unpublished). There are also some records (e.g., Lessmark, 1976) that the Fegen spring‐spawners were once more widely distributed compared with the present distribution which is limited to the NE basin (Figure 2), indicating a decline in abundance over time (Thörne & Carlsson, 2004). However, the spring‐spawning population has always been considered rare, and there is not sufficient monitoring data to establish a proportionally stronger decline compared with the sympatric autumn‐spawners.

In this study, we investigated recent phenotypic changes in the sympatric Fegen populations in relation to possible effects of environmental factors. Specifically, we compared historic (1960s) and more recent (1990–2000s) morphological data from Fegen to changes in local temperatures over the same period, discussing a possible eco‐phenotypic response directly or indirectly related to temperature by means of plasticity and (or) microevolution. Microsatellites markers were used to study genetic structure and amounts of gene flow. We further estimated effective population sizes, and searched for signs of genetic bottlenecks, to investigate whether elevated gene flow could potentially explain observed phenotypic changes. The time since divergence between the two Fegen populations was also estimated. By combining data from detailed morphological and genetic analyses with results from previous studies of other sympatric Baltic cisco populations, an overall aim was to gain further understandings of the evolution, maintenance, and possible reasons for recent collapses of these two‐population systems.

## 2 MATERIAL AND METHODS

Lake Fegen (23.5 km^2^) is situated 132 m above the sea level in Southern Sweden (Figure 2). The average water depth is 7.5 m with a maximum of 38 m (NE basin). In addition to Baltic cisco, perch, *Perca fluviatilis*, and roach, *Rutilus rutilus*, represent the most abundant indigenous fish species (Thörne & Carlsson, 2004). These authors listed a total of 19 fish species, including adjacent watercourses emptying in the lake (see Appendix 1). Fegen has a history of fish introductions, including that of pikeperch which led to an established population in the late 1990s (see Appendix 1).

More detailed ecological data on the Fegen cisco populations in relation to, for example, niche segregation, are scanty. Stomach contents in April consisted almost solely of copepods in both populations (Lessmark, 1976). All ciscoes from Fegen show comparatively slow growth and small adult size, for example, compared with ciscoes in the adjacent Lake Kalvsjön. In addition, the spring‐spawners show slower growth than the sympatric autumn‐spawners (Lessmark, 1976). Comparably slow growth has also been noted for bream, *Abramis brama*, a species that usually thrive under rich conditions, which has been interpreted as a sign of relatively poor nutrient status of the lake (Thörne & Carlsson, 2004).

### 2.1 Samples

Primary material consisted of 376 Baltic ciscoes from Fegen collected during the years 1995–2008 (Table 1). Part of this material (*n* = 149) was included in the genetic study by Delling et al. (2014). For comparisons, we also used historical morphological data for 50 spring‐spawners (*SS*) and 100 autumn‐spawners (*AS*) from Fegen collected in 1960–1969 (Svärdson, 1979 table 26, p73). Additional specimens and data from other lakes were used to address certain methodological issues related to comparisons of historical and more recent morphological data (Table A3). All historical data, earlier published only as means and standard deviations by Svärdson (1979), were reanalyzed based on archived individual measurements from original protocols recovered at the Institute of Freshwater Research, Drottningholm.

**Table 1:** Material of *C. albula* from L. Fegen analyzed with microsatellites and morphology, divided on “ripe and running” (*rr*) or ripe (*r*) spring‐ (*SS*) and autumn‐ (*AS*) spawners

Year	Month	*SSrr*		*ASrr*		*SSr*		*ASr*		Uncertain			Total
		f	m	f	m	f	m	f	m	f	m	?	
1995	May	10										10	20
2003	August									11	7		18
2007	November			30	3			16	38				87
2008	April	1	1									1	3
2008	May	12	65			1	8			31	55	2	174
2008	November			30				1	32	1	10		74
Total		23	66	60	3	1	8	17	70	43	72	13	376

Note

“Uncertain” are fish without sex determination (“*?*”) or with gonads with uncertain status. Material from 1995 and 2003 lack *Tl*. The 10 *SSrr* from 1995 listed as females (*f*, determined in the field) were not possible to sex determine after preservation.

The historical material was collected during spawning from “ripe and running” (*rr*) individuals that could be strictly classified as *AS* or *SS* (Svärdson, 1979). The primary recent material includes *AS* and *SS* collected while spawning (*SSrr* and *ASrr*), but also specimens found to be ripe but not running (*SSr* and *ASr*), and some fish with unknown or uncertain gonadal status that were immature and/or collected outside the spawning seasons (Table 1). As detailed below, depending on the analysis or question, different subsets of the recent material were assigned to *SS* or *AS* based on gonadal status (*rr* and *r*) and time of capture, or from DNA (microsatellites) and morphology with the suffix *mg* (morphometry and genetics).

### 2.2 Morphological analyses

Based on Svärdson (1979), the main morphological differences between *AS* and *SS* in Fegen are vertebral counts (*Vc*), eye diameter (*Ed*), and head length (*Hl*), with *SS* possessing fewer vertebrae, a proportionally larger head and larger eye.

Both total length (*Tl*) and standard length (*Sl*) were measured (whereas only *Tl* existed for the historical materials). Sex for mature specimens (*rr* or *r*) was determined in the field or at the laboratory shortly after capture. For the remaining unclassed specimens, sex was determined during final morphological analyses.

The majority of recent specimens were analyzed in a frozen (slightly thawed), fresh condition. *Tl* was measured to the nearest 1.0 mm from the anterior tip of the head (tip of lower jaw in the case of ciscoes) to the posterior tip of the caudal fin, with both fin lobes folded back. *Sl* was measured with a digital caliper to the nearest 1.0 mm from the anterior tip of the snout to the end of the caudal peduncle. Head length (*Hl*) was measured point to point with a digital caliper to the nearest 0.1 mm from the tip of the snout (upper jaw) to the posterior margin of the operculum (the uppermost bone of the three bones making up the functional gill cover).

**Figure 3:**
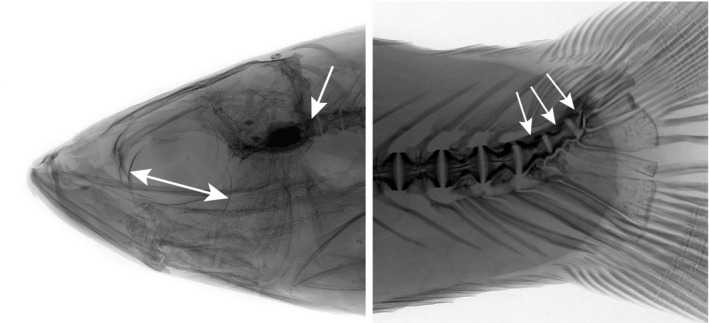
Radiograph images of head and caudal skeleton of *C. albula*, describing measurement of eye diameter, position of the Atlas vertebra, and the three last vertebrae (herein included in total counts)

Measuring the size of the eye (or the eye cavity in a weakly ossified fresh or fixed small coregonid) is notoriously tricky, and repeated trials often result in deviations. However, using X‐ray, the sclera of the eyeball in the anterior and posterior margin of the eye is clearly visible, and the horizontal eye diameter can be measured with a high degree of accuracy, using a reference length for calibration (Figure 3). The number of vertebrae was also counted from digital X‐ray images, starting with the Atlas vertebrae behind the head and ending with the three upturned vertebrae in the caudal skeleton (here counted as three separate vertebrae; Figure 3). In case of occasionally observed vertebrae fusions, the actual number of vertebrae was estimated from the number of neural spins or ribs.

No documentation seems to exist on how morphological measurements and counts were obtained for the historical materials, and no such specimens from Fegen have been preserved. Thus, only original protocols with individual data have been available for the purpose of this study. Besides the variables measured for the recent specimens, the historical materials also included data on gill raker and scale counts, snout length, body depth, snout to dorsal origin distance, and snout to pelvic origin distance (Svärdson, 1979).

**Figure A1:**
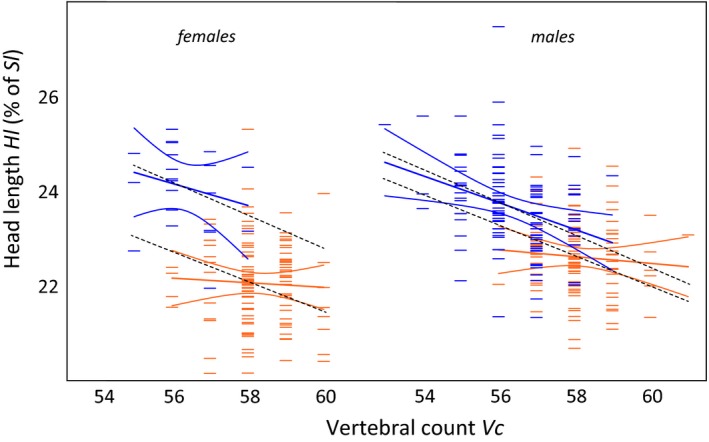
Proportional head length plotted against vertebral counts for specimens of *C. albula* from Fegen. *SSmg* (*n *= 145) in blue, *ASmg* (*n *= 231) in orange. Regression lines with 95% confidence interval given for each group separately. Dashed black lines indicate the hypothetic “segmental effect” for each group separately

During the course of the study, it became obvious that historical and recent morphological data were not fully comparable regarding *Vc* and *Hl*, which most likely reflects systematic methodological differences. Due to a lack of historical preserved material from Fegen, we measured and counted these variables for preserved historical material from Ören and compared those results with published data from the same populations and time period (Svärdson, 1979). As detailed in Appendix 1 (Table A4 with associated text), this allowed for adjustments so that proper comparisons between recent and historic data were possible. In brief, we added 1.36 vertebrae per fish and reduced head lengths by *c*. 1% (in proportion to *Tl*) in the historic data. The relation between *Vc* and *Hl* was also investigated to confirm that the larger head in *SS* was not the result of a proportionally shorter body due to fewer vertebrae (Figure A1).

**Figure 4:**
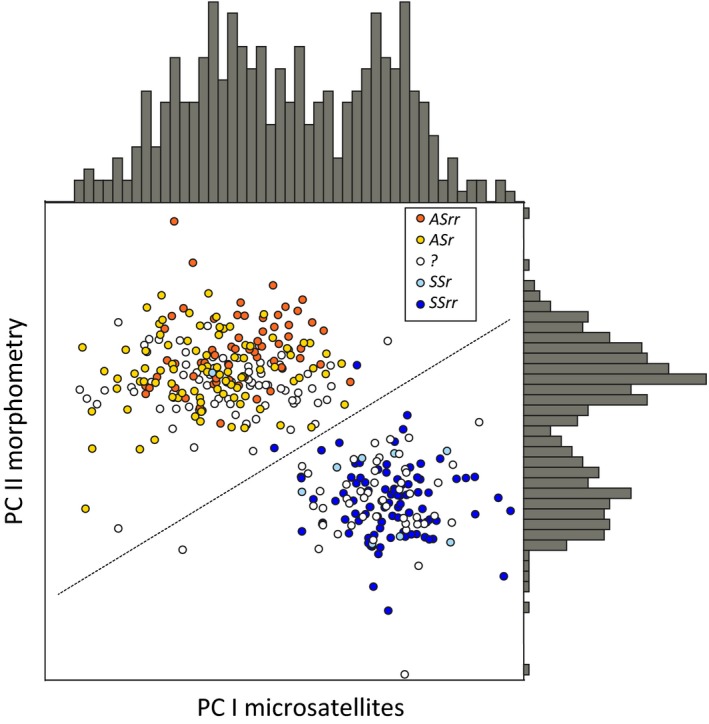
Morphometric PC II plotted against genetic PC I for the entire recent material of *C. albula* from Lake Fegen (*n *= 376). Frequency distributions of PC scores are shown on the opposite axes. The dashed line indicates the selected division into *ASmg* (upper left) and *SSmg* (lower right). See text for details. Filled symbols indicate independent assignment to *AS* or *SS* as ripe (*r*) or ripe and running (*rr*) determined in relation to date of capture. Unfilled symbols represent specimens not possible to assign to *AS* or *SS* based on gonads

**Figure A2:**
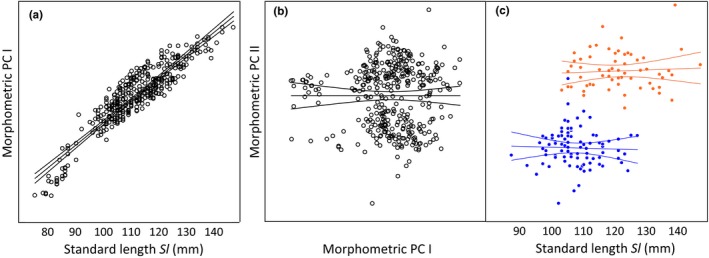
Morphometric variation in recent material of *C. albula* from Fegen. (*n *= 376): (a) PCI plotted against standard length. (b) PC II plotted against PC I. (c) PC II plotted against PC I for recent *rr* specimens of *C. albula* from Fegen. *SSrr* (*n *= 89) in blue, *ASrr* (*n *= 63) in orange. Regression lines with 95% confidence interval given for total sample (a and b) and for each group separately (c)

Variation in *Hl*, *Ed*, and *Sl* were subjected to a principal component analysis (PCA) using the software SYSTAT13. Comparing the resulting principal components to standard lengths, showed that the second component (PC II) could be treated as a size‐independent morphometric “shape” variable (Figure A2; Table A5). The morphometric PC II was used in combination with a corresponding “genetic component” based on factorial correspondence analysis on microsatellite genotypes (details below), to ordinate the total recent material. This resulted in two virtually nonoverlapping clusters, which could be divided into two groups by addition of a (somewhat arbitrarily placed) straight line (Figure 4).

We compared historic data on eye diameter (*Ed*) from Svärdson (1979) with recently collected specimens classified as *rr* or *r*. Potential temporal changes in relative *Ed* within SS and AS, respectively, were tested for with ANCOVAs, using *Hl* as covariate to account for allometric growth. Since the historical specimens were larger on average, the recent material was restricted to specimens with *Hl* > 25.5 mm to allow for comparison of similarly sized fish (after exclusion of small recent specimens, no significant difference in *Hl* between historical and recent specimens remained within AS and SS; *t* test: *p *= .44 for *SS*, *p *= .62 for *AS*).

**Figure A3:**
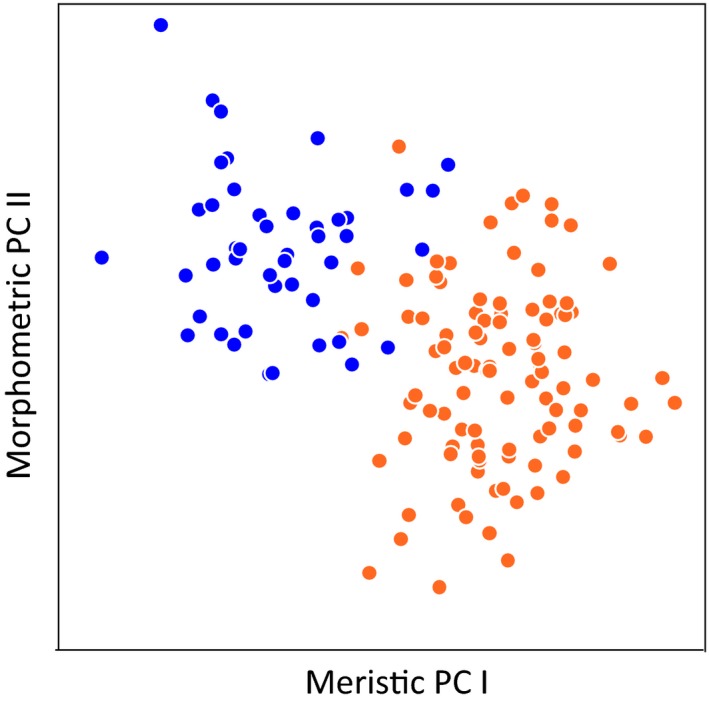
Morphometric PC II plotted against meristic PC I for historical *rr* specimens of *C. albula* from Fegen. (Fegen *SSrr* and *ASrr* in Table A1). *SSrr* (*n *= 45) in blue, *ASrr* (*n *= 100) in orange

To study the degree of morphological distinction among historic *SSrr* and *ASrr*, all studied characters from the original protocols (Svärdson, 1979) were also subjected to PCA (Figure A3; Tables A6 and A7). This independent analysis was done to investigate whether adding further morphological characters was sufficient to unambiguously distinguish (i.e., without overlap) the two forms in the historic material.

### 2.3 Molecular analyses

DNA was extracted from fin‐clips preserved in 95% EtOH, and all individuals were genotyped for the same set of nine microsatellites as in Delling et al. (2014). We refer to that paper for details on markers and laboratory procedures (DNA extraction, PCRs).

A factorial correspondence analysis (FCA) was performed with genetix 4.05.2 (Belkhir, Borsa, Chikhi, Raufaste, & Bonhomme, 2004) to visualize relative similarity among individual multilocus genotypes. fstat 2.9.3.2 (Goudet, 1995) was used to compute unbiased estimates of expected heterozygosity, allelic richness, and *F*‐statistics, and to evaluate deviations from Hardy–Weinberg proportions and genotypic equilibrium.

structure 2.3.4 (Falush, Stephens, & Pritchard, 2003; Pritchard, Stephens, & Donnelly, 2000) was employed to identify the most likely number of genetic clusters (*K*) in the total material. *K* was increased from 1 to 5, with true *K* inferred following Evanno, Regnaut, and Goudet (2005) as implemented in structure harvester (Earl & vonHoldt, 2012). structure was run without prior information on sampling or phenotypic information, assuming admixture and correlated allele frequencies between clusters. As recommended by Gilbert et al. (2012), we used 20 replicate runs per *K* with a burn‐in of 100,000 steps followed by 200,000 MCMC replicates.

To identify putative non‐neutral outlier loci that can yield biased parameter estimates in certain analyses, we used LOSITAN (Antao, Lopes, Lopes, Beja‐Pereira, & Luikart, 2008) that implements the *F*_ST_ simulation approach by Beaumont and Nichols (1996). Signs of genetic bottlenecks where tested for with bottleneck 1.2.02 (Piry, Luikart, & Cornuet, 1999), applying the two‐phase mutation model (Cornuet & Luikart, 1996) with settings recommended for microsatellites (i.e., 95% single‐step and 5% multistep mutations and a variance of 12 for multisteps mutations; Piry et al. (1999)). Results were evaluated with a one‐tailed Wilcoxon test for heterozygote excess (i.e., if present expected heterozygosity was higher than the one expected at mutation‐drift equilibrium) using 10,000 replicates. Using the procedure implemented in bottleneck, we also evaluated the presence of allele frequency mode shifts, as expected following a reduction in effective population size (Luikart & Cornuet, 1998). To search for indications of bottlenecks, we further computed a modification of Garza and Williamson's (2001) *M*‐ratio as implemented in arlequin 3.5 (Excoffier, Laval, & Schneider, 2005).

Contemporary gene flow and migration between sympatric spring‐ and autumn‐spawning populations were assessed with bayesass 3.0 (Wilson & Rannala, 2003). Each simulation was run for 10 × 10^6^ iterations sampled every 2000 step, with the first 3 × 10^6^ iterations omitted as burn‐in. As recommended by Meirmans (2014), we used several (5) independent runs with different random seeds and calculated Bayesian deviance (Faubet, Waples, & Gaggiotti, 2007) to evaluate differences in convergence among runs.

Historical demographic parameters were assessed under an “Isolation with migration model” (Nielsen & Wakeley, 2001) using an ABC‐approach (Approximate Bayesian Computation) with popabc (Lopes, Balding, & Beaumont, 2009). Under this model, two subpopulations, diverged from a common ancestral population sometime in the past, may be connected by gene flow in both directions. Prior distributions for demographic parameters are listed in Table A8. Similar to in Lopes and Boessenkool (2010), mutations simulated for the microsatellites followed a stepwise model with rates drawn from a lognormal distribution of base 10 (prior) with a mean (−4) sampled from a normal distribution (hyper‐prior) and a standard deviation set to a fixed value (0.5). A total of 10 × 10^6^ data sets were simulated with popabc. Out of these, 1,000 were retained (0.0001 rejection level) based on their closeness to the observed data at a set of 17 “summary statistics” (heterozygosity, variance in allele length, number of different alleles, kurtosis of allele's lengths, Shannon's diversity index, heterozygosity‐based Nm estimator) computed within subpopulations and for the total material. Posterior parameter estimates with associated probability densities were assessed using nonlinear regression (neural network method adjusted for heteroscedasticity in R‐package “abc”; Csilléry, François, & Blum, 2012).

## 3 RESULTS

As detailed below, ciscoes in Fegen fall into two forms according to (a) spawning time, (b) morphology, and (c) nuclear genetic markers, but none of these independent data sets can alone be used to divide all fish into well‐separated groups. Combining morphologic and genetic data, however, significantly enforced the distinction between the two forms (Figure 4), and since information on spawning time was missing for parts of the material, the below results are mainly based on this joint morphologic–genetic assignment (*ASmg*, *n *= 231, and *SSmg*, *n *= 145). Notably, only three out of 248 specimens sampled as ripe and running (*rr*) or ripe (*r*) deviated from its expected group, according to the division based on independent morphologic–genetic data (Figure 4).

### 3.1 Morphology

**Figure 5:**
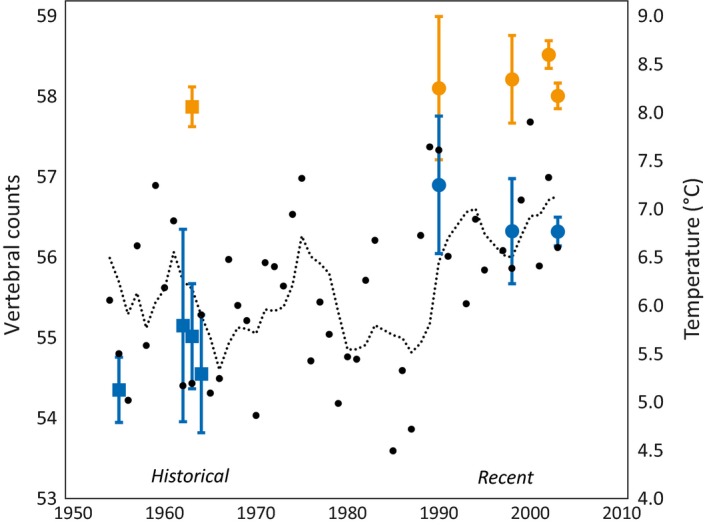
Variation over time for vertebral counts in 145 historical (pre 1970) and 376 recent (after 1990) *C. albula* from L. Fegen (*AS* in orange, *SS* in blue). Counts (adjusted with +1.36 vertebrae for historical data) are given as mean ± 1.96 *SE*. Black dots show annual average mean air temperatures (with 5‐year sliding average) from a nearby meteorological station. Note that vertebral counts have been displaced 5 years earlier (from the year of sampling; cf. Tables 1 and A3) to roughly correspond to the time of early development of the adult specimens

**Figure 6:**
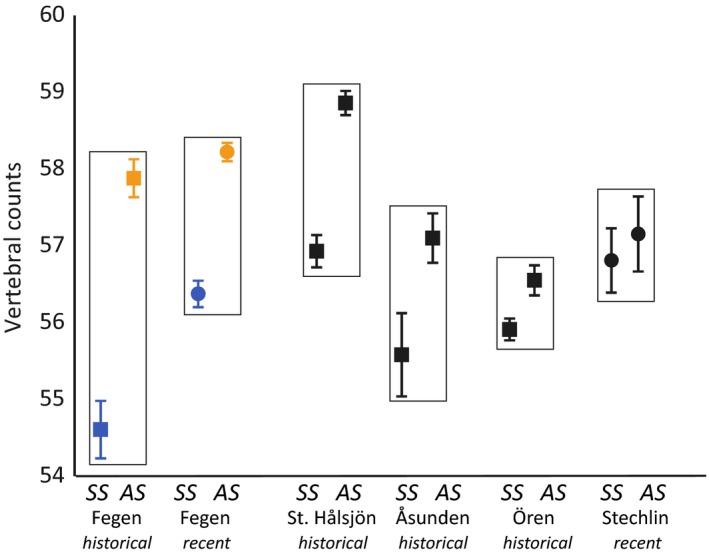
Comparison of average vertebral counts (±1.96 *SE*) for different sympatric population pairs of *C. albula*. Counts were adjusted for historical data (+1.36 vertebrae) from lakes Fegen (*n *= 145), St. Hålsjön (*n *= 277), Åsunden (*n *= 98), and Ören (*n *= 377). Counts for Lake Stechlin (*n *= 34) were taken on recent material in the NRM collection (NRM 52628 and 52629)

A comparison of recent and historical vertebral counts confirmed that *SS* have significantly fewer vertebrae than *AS*, with just minor year‐to‐year variation within each group (Figure 5). In contrast, no such differences among recent *rr* and *mg* individuals within *SS* and *AS*, respectively, could be detected (not shown). Comparing vertebral counts from several lakes showed that *SS* often have lower counts compared with sympatric *AS*, but that this difference was most prominent in Fegen (Figure 6).

**Figure A5:**
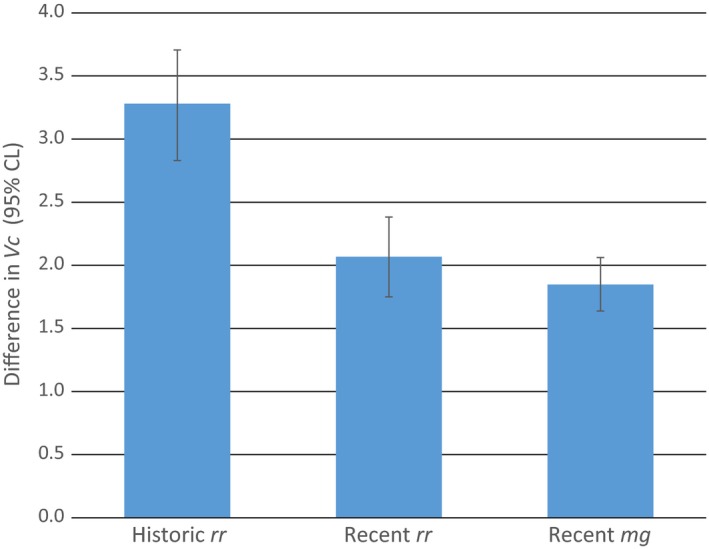
Difference in average vertebrae counts between autumn and spring‐spawning individuals in Fegen with 95% confidence intervals (based on 10,000 resamplings)

The average number of vertebrae in *SS* increased over time, whereas no such change seemed to have occurred in *AS* (Figure 5). As a consequence, the absolute difference in average number of vertebrae between the two groups decreased over time; from about 3.3 vertebrae in the 1950–1960s to about 1.5 in the 1990–2000s (Figure 5, Figure A5). Over the same time period, average local air temperature increased significantly from 6.0°C in the 1950–1960s to 6.8°C in the 1990–2000s (*t* test: two‐sided *p *= .002, *df* = 30), and there exists an apparent correlation between the increase in air temperature and that for *SS* vertebral counts (Figure 5). Anomalies in the vertebral column, mainly vertebrae fusions, were noted in 27 specimens (8 *ASmg* and 19 *SSmg*), that is, more commonly in *SS* (13.1%) compared with *AS* (3.5%) (Fisher's exact test: two‐sided *p *< .001).

**Figure 7:**
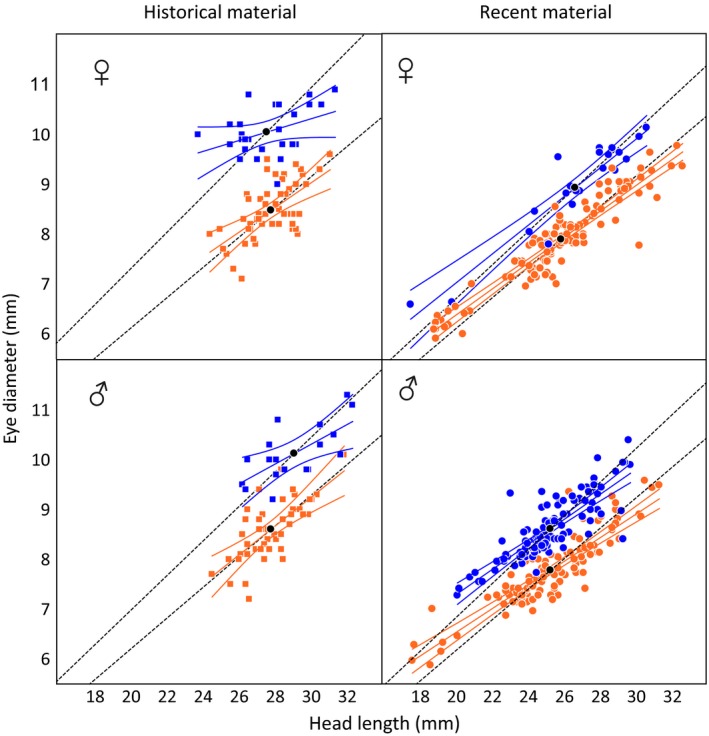
Eye diameter plotted against head length for historical (*n *= 145) and recent (*n *= 376) material of *C. albula* from Lake Fegen, divided by sex (*AS* in orange, *SS* in blue). Linear regression lines with 95% confidence bands are shown for each group separately. The dashed black lines show hypothetical isometric growths based on average eye diameters and head lengths (black dots). Head lengths in the historical material have been slightly adjusted (0.938 × *Hl*) to allow comparisons (see text for details)

**Figure 8:**
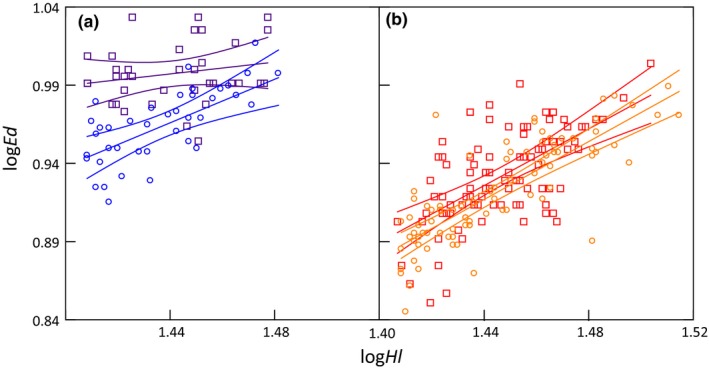
Eye diameter log*Ed* plotted against head length log*Hl* for specimens with *Hl* > 25.5 mm. Linear regression line with 95% confidence interval is shown for each group separately: (a) historic *SS* (indigo squares, *n *= 36) and recent *SSrr* and *r* (blue circles, blue, *n *= 95). (b) historic *AS* (red squares, *n *= 39) and recent *ASrr* and *r* (orange circles, *n *= 87)

**Figure A6:**
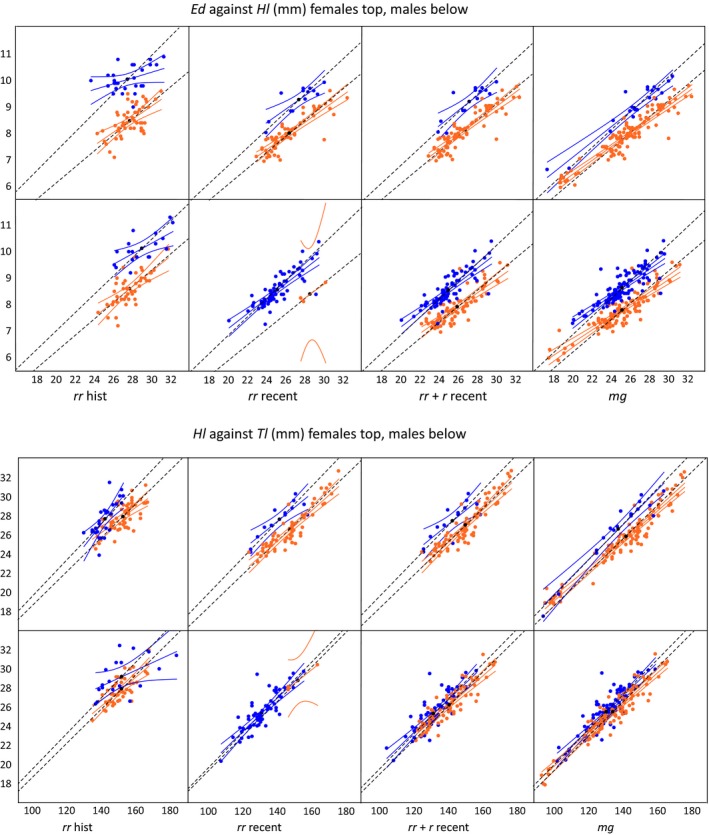
Eye diameter plotted against head length (upper panels) and head length plotted against total length (lower panels) for historical (*n *= 145) and recent (*n *= 376) materials of *C. albula* from Fegen, divided by sex (*AS* in orange, *SS* in blue). Linear regression lines with 95% confidence bands are shown for each group separately. The dashed black lines shows hypothetical isometric growths based on average eye diameter, head length, and total length (black dots). Head lengths in the historical material have been slightly adjusted (0.938 × *Hl* = *Hla*) to allow direct comparisons (see “Comparison of methods to obtain morphological data”). Note that four of the graphs are also shown in Figure 7 (*Ed* against *Hl* for *rr* historic and *mg* recent)

A series of biplots (Figure A6) on morphometric characters (*Hl* against *Tl* and *Ed* against *Hl*) for the recent and historical materials separately showed a trend toward less distinction between *SS* and *AS* for *Ed* over time, particularly in females (Figure 7). Plotting *Ed* against *Hl* (both log‐transformed) for both sexes combined revealed a marked temporal difference in *SS* but just a minor difference in *AS* (Figure 8). Tests for homogeneity of slopes confirmed a significant difference for *SS* but not for *AS* (*SS* *p* = .02, *AS* *p* = .94). Parallelism in the *AS* data set, allowing for ANCOVA, indicated a small but significant difference in *Ed* over time (*p *= .006). It is, however, hard to judge whether this is a true difference or an artifact related to the adjustment needed for the historical *Hl* data (See Appendix 1). Different slopes in historic and recent *SS* (Figure 8a) precluded ANCOVA. However, within the studied *Hl*‐range, a *t* test for *Ed* was highly significant (*p *< .001) revealing a true change in *SS* eye diameter over time.

### 3.2 Molecular data

**Figure A7:**
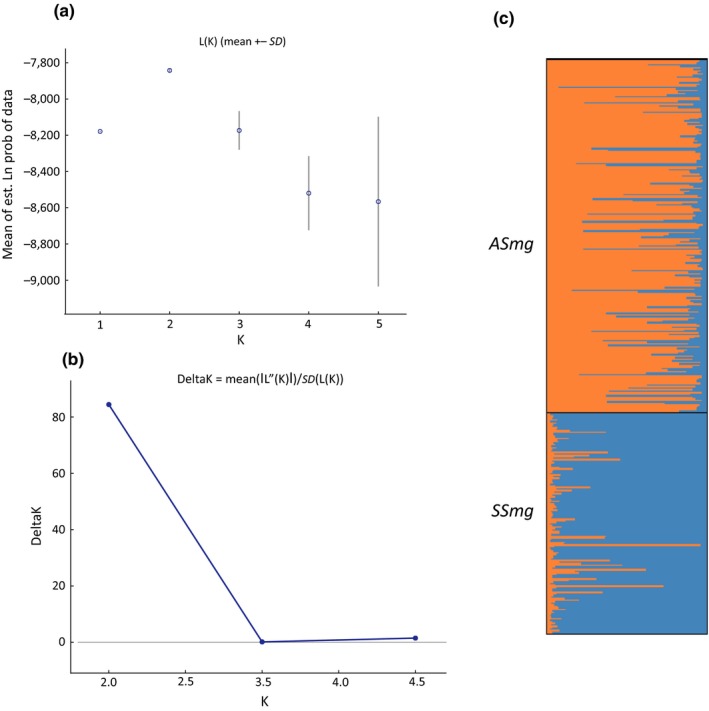
Results from analyses with STRUCTURE (9 microsatellites, *n *= 376): (a) Ln(prob.) of the data at 1–5 genetic clusters (*K*) with (b) corresponding values of Delta *K* (Evanno et al., 2005). The colored bars in (c) illustrate inferred ancestry at *K* = 2 for single individuals in the two clusters identified from a combination of morphologic and genetic data (above/below line in Figure 4)

The analysis with structure based only on microsatellite genotypes yielded highest likelihood for two genetic clusters (*K* = 2; Figure A7). No statistically significant deviations from expected Hardy–Weinberg proportions or pairwise genotypic equilibrium occurred within *SSmg* and *ASmg*, whereas a significant heterozygote deficiency across loci existed in the total material (Table 2).

**Table 2:** Genetic variation in autumn‐ (*n* = 231) and spring‐spawning (*n* = 145) *C. albula* from Lake Fegen (cf. Table 1): number of alleles observed (*N_A_*); allelic richness (*AR*) based on 143 diploids; expected heterozygosity (*H*_E_); Garza–Williamson's ratio (*M*)

Locus	*N*_A_			*AR*			*H*_E_			*M*		*F*_IS_			*F*_ST_
	*ASmg*	*SSmg*	Total	*ASmg*	*SSmg*	Total	*ASmg*	*SSmg*	Total	*ASmg*	*SSmg*	*ASmg*	*SSmg*	Total	
*Cisco9*	12	8	12	10.9	8.0	10.0	0.52	0.50	0.51	0.52	0.35	0.03	0.03	0.04	0.02***
*Cisco1*	4	2	4	3.6	2.0	3.4	0.39	0.50	0.45	0.27	0.13	−0.01	0.14	0.10*	0.09***
*Str73*	2	1	2	1.9	1.0	1.8	0.01	0.00	0.01	0.67	0.33	0.00	n.a.	0.00	0.00
*BWF2*	23	14	25	20.6	13.9	19.9	0.59	0.47	0.55	0.24	0.14	0.01	−0.02	0.01	0.01***
*Sfo23*	35	22	35	33.0	22.0	32.7	0.95	0.90	0.95	0.45	0.29	0.00	−0.01	0.01	0.03***
*Cisco1*	4	2	4	3.6	2.0	3.3	0.45	0.49	0.50	0.36	0.18	0.06	0.00	0.10*	0.13***
*BWF1*	27	17	30	24.9	17.0	25.3	0.79	0.71	0.83	0.30	0.19	−0.01	0.05	0.10***	0.16***
*Sfo8*	5	4	5	4.6	4.0	4.8	0.45	0.42	0.44	0.38	0.31	−0.04	−0.03	−0.04	0.00*
*Cocl23*	5	2	5	4.6	2.0	4.2	0.31	0.28	0.30	0.38	0.15	−0.01	0.01	0.01	0.02***
Average (9 loci)	13.0	8.0	13.6	12.0	8.0	11.7	0.50	0.47	0.50	0.40	0.23	0.00	0.02	0.04***	0.07***
Average (8 loci)	11.3	6.9	11.5	10.4	6.9	10.0	0.46	0.44	0.46	0.41	0.24	0.01	0.01	0.03*	0.04***

Note

*F*_IS_ quantifies deviations from Hardy–Weinberg proportions and *F*_ST_ measures population divergence. Averages based on 8 loci were calculated without the outlier marker *BWF1* (see text for details).

Abbreviation: n.a., not applicable.

* *p* < .05.

*** *p* < .001.

The average number of alleles observed, allelic richness, and expected heterozygosity was higher in *ASmg*. Among 122 alleles in total, 55 were unique to one of the groups (50 and 5 private alleles in *ASmg* and *SSmg*, respectively). Significant allele frequency differences among *SSmg* and *ASmg* occurred at all but one locus and in total, with an overall *F*_ST_ of 0.07 (Table 2). In contrast, no allele frequency differences could be detected between *ASmg* individuals collected from the three main lake basins (not shown).

**Figure A8:**
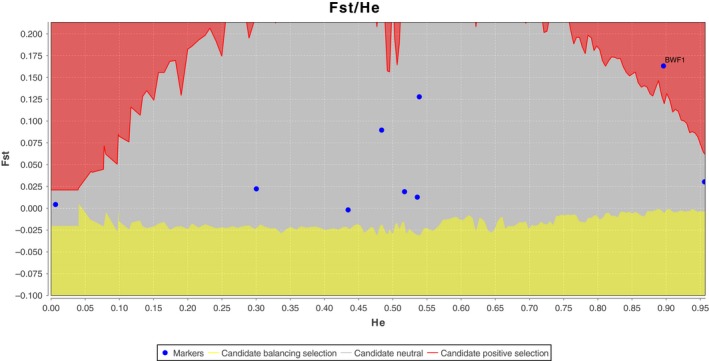
Graphical depiction of result from analysis with LOSITAN (cf. Table A9)

**Figure A9:**
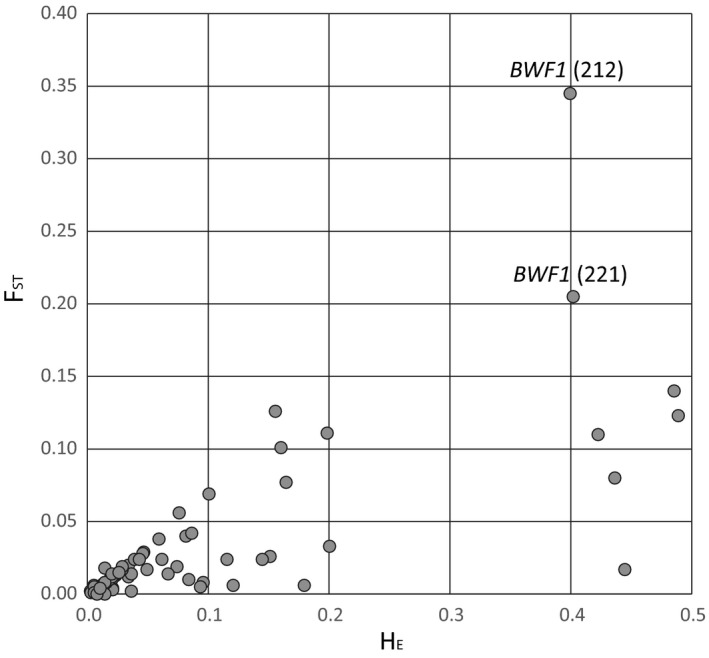
*F*_ST_ per allele (between *SSmg* and *ASmg*) versus *H*_E_, with the two most divergent alleles highlighted (both at BWF1, cf. Figure A8)

One locus (*BWF1*) was identified as a putative outlier, displaying higher differentiation between *SSmg* and *ASmg* than expected under selective neutrality (*p *= .013; Table A9, Figures A8 and A9). Hence, several of the analyses below (assuming neutrality) were based on eight loci only, to avoid potential bias.

**Figure 9:**
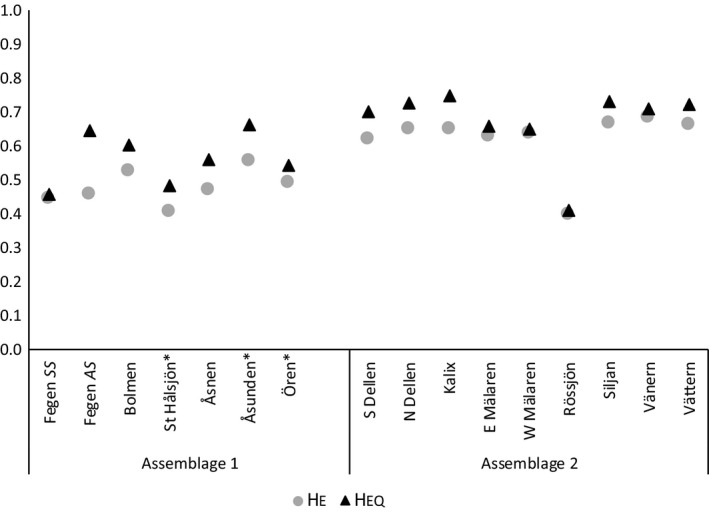
Estimates of current expected heterozygosity (gray circles) and heterozygosity expected under mutation‐drift equilibrium (black triangles) as calculated with BOTTLENECK (two‐phase mutation model, 8 microsatellites). In addition to the Fegen ciscoes, autumn‐spawners from other Swedish lakes and Baltic Sea (Kalix) divided into two population assemblages (cf. Delling et al., 2014) are included for comparison. Asterisks mark lakes previously inhabited by spring‐spawners

Signs of a genetic bottleneck were seen for spring‐spawners. The Garza–Williamson M‐ratio (Table 2) was consistently lower in *SSmg* than in *ASmg* (paired *t* test: *p *= .001). Observed gene diversity (*H*_E_) in both populations was found to be lower than the levels expected under mutation‐drift equilibrium (*H*_EQ_), that is, a pattern in the opposite direction as anticipated for populations following a genetic bottleneck where *H*_EQ_ is expected to be reduced faster than *H*_E_ (Cornuet & Luikart, 1996). A significant difference between *H*_E_ and *H*_EQ_ was observed for *ASmg* (0.46 vs. 0.65, *p* = 1.00), whereas *H*_E_ and *H*_EQ_ were not significantly different in *SSmg* (0.44 vs. 0.46, *p* = .77; Table A10). Expanding the bottleneck analysis to include 14 additional populations from Delling et al. (2014) revealed that *H*_E_ < *H*_EQ_, for unknown reason(s), is a general pattern in Swedish ciscoes (Figure 9, Table A10). Thus, if the Fegen *SS* population originally displayed higher *H*_EQ_ (more alleles than at present), the observation of *H*_E_ ≈ *H*_EQ_ is actually in line with what to be expected following a recent bottleneck.

The five runs with bayesass (8 loci) yielded almost identical parameter estimates and Bayesian deviances, and all chains were therefore merged before final analysis using tracer v. 1.6 (Rambaut, Drummond, Xie, Baele, & Suchard, 2018). Contemporary levels of gene flow into *ASmg* and *SSmg* were estimated to less than 0.01 per generation with a point modal estimate for immigration into *AS* (from *SS*) being twice as high as in the opposite direction (0.008 vs. 0.004) although with highly overlapping probability intervals (Table 3).

**Table 3:** Estimates of immigration rates (*mig*), effective population size (*N*_E_), and time since divergence (*t*) from bayesass (contemporary *mig*) and popabc (long‐term *mig*, *N*_E_ and *t*) based on 8 loci

	Mode estimate (95% probability interval)
Contemporary immigration (BayesAss)	
Into *AS* (from *SS*)	0.008 (0.000–0.039)
Into *SS* (from *AS*)	0.004 (0.000–0.020)
Long‐term immigration (PopABC)	
Into *AS* (from *SS*)	0.007 (0.000–0.021)
Into *SS* (from *AS*)	0.003 (0.000–0.020)
Long‐term effective population size (PopABC)	
Ancestral population	25,168 (13,813–31,702)
*AS*	4,305 (2,023–5,272)
*SS*	189 (0–1,577)
Long‐term effective migrants per generation (PopABC)	
Into *AS* (from *SS*)	30.1
Into *SS* (from *AS*)	0.6
Time since divergence (PopABC)	
Generations	486 (88–1,467)
Years (assuming *G* = 4)	1,944 (352–5,866)

Note

Point estimates of effective number of migrants per generation were obtained by multiplying corresponding estimates of long‐term *mig* and *N*_E_.

Historical (long‐term) estimates of immigration rates obtained with popabc (8 loci) under an isolation with migration model were almost identical to the contemporary estimates (Table 3). The estimated historical effective population size was significantly lower in *SSmg* (*N*_E_ = 189, 95% PI: 0–1,577) compared to *ASmg* (*N*_E_ = 4,305, 95% PI: 2,023–5,272). Time since divergence was estimated to 486 generations (95% PI: 88–1,467) corresponding to 1,944 years (PI: 352–5,866; Table 3), assuming a generation interval of 4 years (Delling et al., 2014).

## 4 DISCUSSION

Our results reveal rapid phenotypic change in the spring‐spawning (*SS*) Fegen population, manifested as an increased number of vertebrae and a decreased relative eye size from the 1960s to the 1990s. In contrast, the sympatric autumn‐spawning (*AS*) population has remained virtually unchanged. Analyses of microsatellite data show lower allelic richness and signs of a genetic bottleneck in the SS population. The same genetic data further reveal a clear difference in long‐term average effective population size (*N*_E_) between the sympatric populations (*SS* < *AS*) and low levels of gene flow in both directions.

As shown in Figure 4, there is not a perfect match between the division of the material into two groups based on spawning time and combined morphologic–genetic data, respectively. Similar incongruences between morphologically defined groups and spawning time were also revealed in the historic data (Figure A3; Tables A6 and A7). Consequently, even if we use vernacular names stipulating spawning period for the two sympatric forms, we conclude that some exceptions are expected between assignments of individuals based on different types of data. Below we discuss different possible explanations for the rapid morphological change seen in Fegen, followed by comments on evolution and disappearance of sympatric Baltic cisco populations in general.

### 4.1 Eco‐phenotypic response to temperature?

Although fluctuations between years in average air temperature close to Fegen (Figure 2) have been extensive (*c*. 4.5–8.0°C), it is striking that after 1990 there are no annual means below 6°C (Figure 5). Phenotypic traits in fish are typically sensitive to abiotic factors, and temperature variation during early development can result in nongenetic changes in many traits. Lindsey (1988) reviewed factors controlling meristic variation in fish. Of the studies reviewed, the data on brown trout, *Salmo trutta*, vertebral counts (*Vc*) in relation to temperature (Tåning, 1952), are probably most relevant for comparison with our results (i.e., a salmoniform fish with similar temperature preferences and vertebral counts as *C. albula*). Tånig's results showed that it required an increase of as much as 5°C to increase the average *Vc* in trout by one. Lowest counts were found around 6°C, and a decrease in temperature also resulted in a slight increase of vertebral counts in six out of eight crosses, that is, a so‐called V‐shaped response (Lindsey, 1988). However, among the studies cited by Lindsey, there was no overall trend for a positive or V‐shaped response in *Vc* from temperature; five out of nine salmonid taxa (*Oncorhynchus* spp., *Salvelinus* spp. and *Salmo salar*) rather showed a negative response.

Runnström (1941) studied water temperature and spawning periods for the sympatric populations in Stora Hålsjön. He concluded that *AS* spawned at around 6°C during the autumn circulation whereas *SS* spawned at about the same temperature during early stratification after the spring circulation. Early development and hatching take place in late spring and early summer in both forms, although a narrower first growth zone on the scales indicates that *SS* hatch somewhat later than *AS* (Svärdson, 1979). Even if more rapid temperature increases in spring could have increased *Vc* in *C. albula*, this would likely have affected *AS*, that spawns in shallow waters, even more (compared with *SS* that spawns below the thermocline where temperatures remain more stable). Furthermore, assuming that *Vc* in *C. albula* responds to temperature much more strongly than in *S. trutta*, one would also have expected larger variation between years within both populations. In conclusion, when comparing to studies of other species, the increase seen for *Vc* in Fegen *SS* (*c*. 1.8) appears much too large to be explained by an increase in air temperature of just about one degree (acknowledging that the relationship between air and water temperatures is complex; see below).

### 4.2 Microevolutionary change?

The alternative to effects of temperature on phenotypic traits in Fegen *SS* is microevolution involving natural selection, random genetic drift, and/or increased gene flow from *AS* into *SS*. Leary, Allendorf, and Knudsen (1985) reported high heritability for *Vc* and other meristic traits in rainbow trout (*Onchorynchus mykiss*), suggesting that genetic change could indeed explain the increase in *Vc* for *C. albula*. However, we are not aware of any similar heritability studies with respect to eye diameter (*Ed*).

For three‐spined stickleback, *Gasterosteus aculeatus*, evolutionary rapid phenotypic changes explained by altered natural selection regimes have been documented (e.g., Marques et al., 2016). Further, Bhat et al. (2014) showed that invasion of Baltic cisco into a typical Fennoscandian two‐population system of whitefish *Coregonus* sp. (planktivorous and benthivorous) led to breakdown of reproductive isolation between the two whitefish forms that collapsed into a more morphologically and genetically homogenous population within just 15 years. Thus, some kind of microevolutionary explanation to the present observations appears feasible.

However, in Fegen, a potential scenario involving natural selection raises several questions. It is unclear to what extent the observed phenotypic differences between *SS* and *AS* are in fact adaptive and related to ecological niche separation. There is a tendency with lower vertebrae counts in *SS* for all studied population pairs (Figure 6). A plausible explanation for this general pattern may involve competition between juvenile *AS* and *SS*, where allocation of growth in *SS* from body segments (vertebrae) into a larger head and feeding apparatus might give an advantage for the offspring when competing with *AS* that hatches somewhat earlier in spring. According to Figure 2 in Airaksinen (1968), it seems that *SS* in Lake Ännättijärvi had larger heads than their sympatric *AS*, even though not clearly stated by the author. Among the four known population pairs in Sweden, however, a larger head in *SS* is only found in Fegen, whereas *SS* in Ören and Åsunden (no data for St Hålsjön) on average displayed *c*. 1% smaller heads in relation to *Tl*, compared with their sympatric *AS* (Svärdson, 1979).

Eronen and Lahti (1988) studied the life cycle of the “winter‐spawning” (March) cisco in the Finnish Lake Kajoonjärvi. They suggested that low fecundity combined with large and protein‐rich eggs could be adaptions related to later hatching and competition with sympatric *AS*, although they only compared data for *AS* from other lakes. Unfortunately, no comparable data on fecundity or egg size exist for the Fegen populations.

Larger eyes are commonly found in fishes in deeper water, although this pattern could partly reflect differences in growth rates (Pankhurst & Montgomery, 1994). Even if most spring‐spawning cisco populations have been reported to spawn deeper than their sympatric autumn‐spawners, (e.g., Svärdson, 1979) little is known regarding their spatial separation outside the spawning seasons. We also note that *SS* have been reported to have significantly larger eyes than their sympatric *AS* (cf. head length above) in only two cases (Fegen and Ännättijärvi) (Airaksinen, 1968; Svärdson, 1979).

A negative relationship between relative eye size and individual growth rate has been reported for *O. mykiss* (Pankhurst & Montgomery, 1994). However, the pronounced eye size difference in Fegen (Figures 1 and 7) is coupled with just a slightly lower growth rate in *SS* (Lessmark, 1976). This may suggest that genetic factors are involved and that local conditions in Fegen (and Ännättijärvi) could have promoted larger eyes in *SS*, possibly in combination with a longer time of divergence than in other lakes with less distinct sympatric populations (see below). Taken together, however, even if natural selection could be responsible for the phenotypic differences between *SS* and *AS*, it seems unlikely that a marked and sudden change in the selection regime may have caused the observed rapid retrogression of *SS* into a more *AS*‐like morphology.

The present microsatellite data indicates a “genetic bottleneck” in *SS* (Figure 9, Table A10). Elevated genetic drift during such a decrease in effective population size is expected to affect the whole genome, which may create random phenotypic change (Falconer & Mackay, 1996). However, to disentangle the relative effect of drift and selection on phenotypic trait means requires experiments in controlled environments (e.g., Rogell et al., 2013). Furthermore, it has not been possible to date the present bottleneck event and to check whether it occurred between the collecting dates of the historical and recent data sets or earlier, although the lake history and some results obtained here indirectly support a more recent bottleneck. From the 1950 to 1960s and onwards, Fegen has suffered from acidification, slight eutrophication, and introduction of pikeperch (Thörne & Carlsson, 2004). Results from gill‐netting surveys also indicate a decline in overall cisco abundance in more recent years (Linderfalk, 2014). Signs of retarded growth (Figures 7 and 8) and a higher frequency of vertebral anomalies in recent *SS* compared with *AS* provides further indirect indications that a declining *SS* population may be subject to unfavorable conditions.

Another not exclusive explanation for rapid phenotypic change could be a recent and temporary influx of genes from *AS* into *SS*. Our results indicate low and rather similar levels of long‐term and contemporary gene flow (immigration rates) in both directions, despite a larger than 20‐fold difference in long‐term average effective population sizes (Table 3). Converting these estimates into corresponding numbers of effective migrants results in about 50 times more migrants from the small SS into the larger AS population than vice versa. Although this estimate is statistically uncertain, it may reflect that autumn‐spawning is the ancestral condition (Delling et al., 2014) and that spring‐spawners more commonly revert into “normal” autumn‐spawning. At the same time, we note that among the 376 specimens included there are three individuals, all autumn‐spawners according to morphology and microsatellites, that seemingly spawned at the “wrong time” (Figure 4), possibly indicating a higher current gene flow from *AS* into *SS*.

Pointing toward a recent boost in gene flow from *AS* to *SS* is also the fact that an earlier allozyme study (30 loci) including *SS* and *AS* from Fegen (Öst, Jansson, & Hamrin, 1990) revealed considerably stronger genetic differentiation (*F*_ST_ = 0.15; 95% CI: 0.10–0.23) than observed herein (*F*_ST _= 0.07; 95% CI: 0.02–0.11). The allozyme data were collected in 1986 and 1989, that is, between the presently analyzed historical and recent materials, where the latter was collected mainly in 2007–2008 (Table 1).

In conclusion, out of the possible microevolutionary explanations for rapid phenotypic change listed above, we consider a temporary period of boosted gene flow from *AS* into *SS* as most likely, possibly associated with the population decline in *SS* manifested as a genetic bottleneck. Among possible explanations for a proportionally stronger decline in *SS*, there is no obvious candidate in the data presented by Thörne and Carlsson (2004), although a slight trend toward increased eutrophication and temporary acidification combined with some changes in the Fegen fish community may have affected Baltic ciscoes negatively. In particular, the establishment of pikeperch during the warmer 1990s (Appendix 1) coincides with the apparent drop in genetic differentiation (*F*_ST_). However, so far pikeperch appears to be relatively scarce, especially in the deeper NE lake basin where the SS population resides (Linderfalk, 2014; Thörne & Carlsson, 2004). Further information or data appear warranted to settle this question more definitely.

### 4.3 Patterns and processes in Baltic cisco evolution

Within a broader time frame, environmental change is probably a key factor behind the evolution, maintenance, and collapses of ciscoes with displaced spawning periods that in several cases has resulted in sympatric populations. Trybom (1903) speculated that the temperature regime in Stora Hålsjön could explain the occurrence of sympatric ciscoes. Svärdson (1979) further argued that autumn‐spawning is an adaptation among “cold water fish species” to maximize their first growth season, whereas spring‐spawning (seen among “warm‐water fishes”) is generally regarded as more beneficial under warmer conditions. This suggestion was in line with his hypothesis of an ancient preglacial origin of the proposed species *C. trybomi* (“spring‐spawning cisco”) during the warmer Eemian interglacial period. However, supported by genetic and geological data, Delling et al. (2014) rather proposed a later and independent postglacial diversification in early‐arriving ciscoes (the so‐called Group I) to higher altitude lakes with past or present sympatric populations, utilizing ice‐dammed lake complexes above the Baltic Ice Lake.

Vuorinen et al. (1981) also found genetic evidence for independent postglacial diversification for cisco populations and further showed that Finnish lakes with spring‐ or winter‐spawners displayed higher water temperatures during winter, compared to other lakes with only autumn‐spawners. These Finnish (and Swedish) lakes are comparatively small, deep and situated at higher altitudes, which results in an earlier development of a permanent ice cover following the autumn circulation. The ice cover, in turn, insulates the lake from continued cooling during wind‐induced circulation (Eklund, 1998, 1999). Higher water temperature during winter was suggested by Vuorinen et al. (1981) to be unfavorable for autumn‐spawners, through elevated egg mortality caused by oxygen deficiency. In line with this hypothesis, a milder climate could be beneficial for autumn‐spawning, especially in smaller high altitude lakes, which indirectly (via intraspecific competition) may be unfavorable for a sympatric spring‐ or winter‐spawning population.

We have estimated the divergence time between *SS* and *AS* in Fegen to a few 1,000 years only, but still the Fegen ciscoes seem to be the most morphologically and genetically differentiated sympatric population pair studied so far. The populations in Lake Ören showed very little morphological differentiation (Svärdson, 1979) and only slight genetic differentiation at 32 allozyme loci (Svärdson, 1988; Vuorinen, 1988). In contrast, the allozyme study including *SS* and *AS* from Fegen (Öst et al., 1990) and our present data have revealed clear differentiation between *AS* and *SS* (see above). In German Lake Stechlin, only subtle morphological differences were found among the sympatric ciscoes and *F*_ST_ estimated across six microsatellites was just 0.004 (Schulz et al., 2006).

Assuming that levels of genetic and morphological differentiation are roughly related to time since divergence, the population pairs in Ören and Stechlin may have evolved much more recently than the more distinct ones in Fegen. Alternatively, the low levels of morphological and genetic divergence could reflect that the “speciation process” has already reversed in those lakes. Regardless, it is obvious that levels of differentiation vary considerably between lakes. In line with an evident independent postglacial divergence, this is also what one could expect; that is, to a certain degree all sympatric population pairs represent individual unique cases.

Comparison of genetic differences between population pairs and estimates of time since divergence using methods and data sets that are not fully comparable (see above) should be done with caution, and so far few such cases in *C. albula* have been studied in detail. However, we suggest that the evolution of sympatric Baltic cisco populations may be a fluctuating process; that is, spring‐spawners may “come and go” as environmental conditions change. More recent and rapid anthropogenic impacts, including slight regional climate change, may have had an overall negative impact on these fragile systems, and it is possible that the declining *SS* population in Fegen has persisted a little longer than in other lakes as it is comparatively old and morphologically distinct, indicating a possible stronger niche differentiation toward *AS*.

Even though we suggest that these sympatric cisco populations could appear almost ephemeral in evolutionary terms, the extinction and decline of *SS* populations in recent decades represent a rapid loss of biodiversity within the far from fully understood *C*. *albula* complex. Further studies of ciscoes from additional Fennoscandian lakes, that is, in Finland, possibly still inhabited by sympatric *C*. *albula* populations, appear urgently needed to gain further knowledge on these interesting populations.

## CONFLICT OF INTEREST

None declared.

## AUTHOR CONTRIBUTIONS

B.D. and S.P. designed the study. B.D performed the morphological work, whereas S.P. analyzed molecular data. B.D. and S.P. performed joint analyses of morphologic–genetic data and drafted the manuscript.

## Figures and Tables

**Figure A4:**
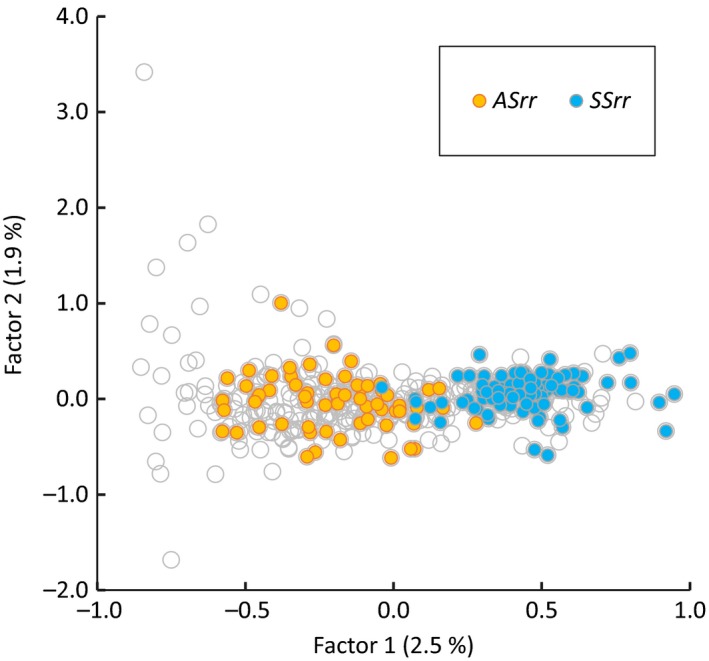
Graphical depiction of individual Baltic cisco genotypes from Fegen following factorial correspondence analysis with GENETIX (9 microsatellites, *n *= 376). *SSrr* (*n *= 89) in blue, *ASrr* (*n *= 63) in orange. Note that scores for Factor 1 are also used in Figure 4 (together with corresponding morphological information)

## Data Availability

The microsatellite data have been deposited at Dryad: https://doi.org/10.5061/dryad.sf7m0cg1k

## APPENDIX 1

### Contents

1. Spawning periods in different cisco lakes (Table A1)
2. Fish fauna in Lake Fegen (Table A2)
3. Comparative material (Table A3)
4. Comparison of methods to obtain morphological data (Table A4)
5. Relation between vertebral counts and head length (Figure A1)
6. PCA for recent material from Lake Fegen (Figure A2, Table A5)
7. PCA for historical material from Lake Fegen (Figure A3, Tables A6 and A7)
8. FCA on multilocus genotypes for recent material from Lake Fegen (Figure A4)
9. Isolation with migration model: Parameters and priors (Table A8)
10. Difference in *Vc* between *SS* and *AS* in historical and recent material (Figure A5)
11. Morphometry of recent and historical material from Lake Fegen (Figure A6)
12. Analysis with structure (Figure A7)
13. Identification of non‐neutral loci (Figures A8 and A9, Table A9)
14. Analyses of genetic bottlenecks (Table A10)

### 1. SPAWNING PERIODS IN DIFFERENT CISCO LAKES

**Table A1:** European lakes where ciscoes with deviating spawning period have been noticed

Country	Lake	Altitude (masl)	Max depth (m)	Area (km^2^)	Spawning period (February to January)												Source
					F	M	A	M	J	J	A	S	O	N	D	J	
Sweden	Ören	196	36	9.2													1
	Fegen	132	36	23.4													1
	Åsunden	164	40	32.7													1
	St. Hålsjön	79	29	3.9													1
Finland	Änättijärvi	190	42	25.1													2
	Sokojärvi	132	40	2.0													2, 3
	Kajoonjärvi	209	40	5.7													2, 3, 4
	Luojärvi	264	12	1.4													2
	Siikajärvi	156		1.8													2
	Kostonjärvi	232	17	44.0													3
	Kokkamo	208		2.0													3
	Pirttijärvi	202		2.0													3
	Suomunjärvi	152		7.0													3
Russia	Small lake in																
	Korpisälke																2
Norway	Varaldsjöen	208		6.0													5
Germany	Breiter Luzin	84	58	3.4													6
	Stechlin	60	69	4.5													6

Note

Yellow, blue, and red refers to populations referred to as “winter‐,” “spring‐,” or “autumn‐spawners” in cited sources. Light blue indicates less common spawning periods for “spring‐spawners.” 1. Svärdson (1979); 2. Airaksinen (1968); 3. Vuorinen et al. (1981); 4. Eronen and Lahti (1988); 5. Huitfeldt‐Kaas (1927); 6. Kottelat and Freyhof (2007).

### 2. FISH FAUNA IN LAKE FEGEN

The information below comes from the extensive data compilation by Thörne and Carlsson (2004) with some additional input from Linderfalk (2014).

**Table A2:** Species composition from survey gill net fisheries in periods 1967–1989 and 1995–2014

Species	Proportion of total number or weight (%)	1967	1980	1983	1989	1995	2003	2014
Baltic cisco	Number	2	4	<1	2	33	40	29
*Coregonus albula*	Weight	<1	1	<1	<1	24	26	10
Perch	Number	25	23	14	28	34	36	34
*Perca fluviatilis*	Weight	21	16	11	32	36	28	27
Roach	Number	64	67	78	56	17	40	14
*Rutilus rutilus*	Weight	54	59	65	31	28	26	23
Ruffe	Number	4	1	<1	2	14	9	8
*Gymnocephalus cernua*	Weight	<1	<1	<1	<1	2	2	2
Pikeperch	Number	0	0	0	<1	<1	1	2
*Sander lucioperca*	Weight	0	0	0	<1	1	11	15
Pike	Number	2	1	2	2	<1	<1	2
*Esox lucicus*	Weight	12	8	2	13	1	6	2
Bleak	Number	<1	0	2	<1	2	3	10
*Alburnus alburnus*	Weight	<1	0	<1	<1	1	2	4
Whitefish	Number	2	3	2	3	<1	<1	<1
*Coregonus* sp.	Weight	6	2	3	<1	2	1	1
Bream	Number	<1	1	<1	5	<1	1	2
*Abramis brama*	Weight	5	<1	5	<1	2	5	11
Burbot	Number	0	0	0	0	<1	<1	<1
*Lota lota*	Weight	0	0	0	0	3	4	6
Rudd	Number	X	X	X	X	<1	0	0
*Scardinius erythropthtalmus*	Weight					<1	0	0
Tench	Number	X	X	X	X	0	0	0
*Tinca tinca*	Weight					0	0	0
Siberian sculpin	Number	0	0	0	0	<1	0	<1
*Cottus poecilopus*	Weight	0	0	0	0	<1	0	<1

Note

Relative abundances are given as percentages of total number or total weight. For the period 1967–1989, very low abundances are noted only as “X”. Note that test fishing methodologies have changed over time, especially between 1989 and 1995. However, on the whole, data are consistent within the two time periods (1967–1989 and 1995–2014), justifying comparisons within (but not between) these periods.

### ADDITIONAL SPECIES NOT INCLUDED IN TABLE A2 ABOVE

White bream, *Blicca bjoerkna*: Two specimens reported in 1967, most certainly misidentification (Linderfalk, 2014).

Brown trout, *Salmo trutta*: A few specimens recorded during electrofishing in inflowing watercourses.

Minnow, *Phoxinus phoxinus*: Recorded during electrofishing in inflowing watercourses, possibly also present on suitable habitats along shore.

European eel, *Anguilla anguilla*: This species is never caught in gill nets, but its regular presence in the lake has been confirmed (see also below).

### HISTORY OF STOCKING AND INTRODUCTIONS

Brook trout, *Salvelinus fontinalis*: Introduced in a few watercourses 1963–1969 and regarded as, at least temporarily, established at one locality in 1967.

Rainbow trout, *Oncorhynchus mykiss*: Stocked in 1965 (100 specimens only), not established.

Tench: Stocked (1930) and established with limited distribution.

Whitefish: Stocked (1942) but probably already present in the lake. The whitefish in Fegen is a typical sparsely rakered form (18–21 gill rakers), most certainly benthos‐feeding.

Pike: Intensive stocking (1939–1961) like in many other Swedish lakes during that period. This kind of stocking of young pike (0+ or 1+) have later been considered as largely inefficient.

European eel: Stocking of eel in Swedish lakes, including Fegen, have a long tradition to compensate for the loss of natural migrating elvers.

Pikeperch: Repeatedly stocked since 1945 but not confirmed as established until in late 1990s.

### 3. COMPARATIVE MATERIAL

**Table A3:** Material of *C. albula* and *C. fontanae* included in comparative morphological analyses

Lake/population	Year of capture	Number of specimens	Type of data/source
Fegen *SSrr*	1960	27	SLU archive
Fegen *SSrr*	1967	10	SLU archive
Fegen *SSrr*	1969	8	SLU archive
Fegen *ASrr*	1968	100	SLU archive
Stora Hålsjön *SSrr*	1939	77	Runnström (1941)^a^
Stora Hålsjön *ASrr*	1940	62	Runnström (1941)^a^
Stora Hålsjön *ASrr*	1953	43	SLU archive
Stora Hålsjön *ASrr*	1968	62	SLU archive
Åsunden *SSrr*	1956	9	SLU archive
Åsunden *ASrr*	1968	89	SLU archive
Ören *SSrr*	1957	100	SLU archive
Ören *SSrr*	1967	102	SLU archive
Ören *ASrr*	1967	75	SLU archive
Ören *ASrr*	1968	100	SLU archive
Ören *SS* uncertain^b^	1958	36	Preserved, NRM 16231–16234
Ören *SSrr* c	1978	11	Preserved, NRM 39286, 39,287
Stechlin *SS C. fontanae*	2005	21	Preserved, NRM 52629
Stechlin *AS*	2005	13	Preserved, NRM 52628

Note

All data from “SLU archive” only consist of individual records from original protocols.

a Mean values with standard deviations (Runnström, 1941).

b Incomplete collecting data but listed as possible PARATYPES in the NRM collection.

c PARATYPES collected during spawning from the same spawning shoal as the HOLOTYPE for *Coregonus trybomi*.

### 4. COMPARISON OF METHODS TO OBTAIN MORPHOLOGICAL DATA

Unfortunately, the historical Fegen material was not preserved. In Svärdson (1979) or in the original protocols, there are no details regarding methods on how morphological measurements and counts where obtained, but likely specimens were examined in fresh condition with vertebrae counted by means of dissection. Similar handwriting on all original protocols from the SLU archive (Table A3) indicates that the same staff took all measurements.

Comparisons between historic records and results from recent analyses of preserved material from Lake Ören revealed systematic differences with respect to number of vertebrae (*Vc*), head length (*Hl*), and eye diameter (*Ed*):

- Historical *Vc* is lower compared with recent data. Possible explanations include (a) vertebrae are more easily counted using X‐ray (as for recent material), (b) uncertainty over whether the Atlas vertebra was included, and (c) uncertainty regarding how the three last “upturned” vertebrae in the caudal skeleton (Figure 3) were counted earlier.
- Historical *Hl* is slightly longer compared to recent data. A possible explanation could be that it was measured from the most anterior tip of head (tip of lower jaw, cf. *Tl* measurements) to most posterior margin of gill cover versus tip of snout to most posterior margin of the operculum. The operculum is the uppermost dermal bone out of three making up the functional gill cover.
- Historical *Ed* is slightly smaller compared to recent data. Possible reasons are mentioned below.

Comparisons of historical and contemporary values (based on preserved historical samples) resulted in slight adjustments of *Vc* (into *Vca*) and *Hl* (into *HLa*). They also elucidated problems comparing different data sets. Details on comparisons are as follows:

*Vertebral counts*

Vertebral counts (*Vc*) were obtained by means of X‐ray from a total of 47 preserved *C. albula* from Lake Ören (Table A3, NRM 16231–16234, 39286, 39287). Out of these, 11 were originally classified as *SS* whereas status (*SS* or *AS*) of the remaining 36 is uncertain. Counts were compared with the historical Ören data set (202 *SS* and 53 *AS*). The difference in *Vc* between historic *AS* and *SS* in Lake Ören is not statistically significant (*t* test) whereas a comparison of all the 47 X‐rayed specimens with the 255 historical ones yielded a difference +1.36 vertebrae (i.e., higher average for the recently analyzed material). Consequently, 1.36 were added to individual vertebrae counts in the historical Fegen material, indicated as *Vca* (adjusted vertebral count). Note that this adjustment is only used in graphic presentations and discussion on trends of changes in *Vc* over time, that is, the correction is not included in any combined dataset that has been analyzed statistically.

*Head length and eye diameter*

In total, 36 *C. albula* (SS/AS) from Lake Ören collected in 1958 were analyzed in the same way as the contemporary material. Measurements included *Tl*, *Hl*, *Hls* (assumed head length, sensu Svärdson (1979), eye diameter measured with a digital caliper (*Edd*) and from X‐ray (*Edx*). Data were compared with historic data for 100 specimens (*SSrr*) collected from Ören in 1957.

Measuring the head sensu Svärdson (1979) (*Hls*) resulted in a *c*. 1% longer head in relation to *Tl*, as compared to *Hl *versus* Tl*. This conclusion is independent of actual status (*SS* or *AS*) of the specimens; that is, this is a strict methodological issue. To make historical data on head length (*Hls*) comparable with recent measurements (*Hl*), historical data for the Fegen material were adjusted as 0.938 × *Hl* = *Hla* (cf. Table A4 where 0.165(*Hl*/*Tl*)/0.176(*Hl*s/*TL*) = 0.938).

Measuring the eye from radiographs gives results comparable to mechanical measurements but with a somewhat smaller standard deviation (Table A4). When comparing historical and contemporary values for *Ed*, there is a weak tendency for larger eyes in the contemporary material irrespective of method. This can represent a true difference, but also a difference in measurement methods. Therefore, we chose not to adjust *Ed* in the historical dataset.

**Table A4:** Average historical and contemporary proportional measurements for preserved material of *C. albula* from Ören

Measurement	*Hls/TL*	*Hl/Tl*	*Edd/Tl*	*Edx/TL*	*Edd/Hls*	*Edx/Hls*
Contemporary (*n* = 36)	0.176	0.165	0.0465	0.0464	0.2637 ± 0.013	0.2632 ± 0.008
Historical (*n* = 100)	0.178			0.0450		

Note

Standard deviation (±) is given for contemporary eye measurements (*Hls* = tip of lower jaw to posterior margin of gill cover, *Edx* = eye diameter obtained from X‐ray).

### 5. RELATION BETWEEN VERTEBRAL COUNTS AND HEAD LENGTH IN *C. ALBULA*

Silfvergrip (1996) used the term “segmental effect” to describe a situation where body length and other morphometric characters are correlated to the number of body segments, that is, vertebrae. Hypothetically, a high number of vertebrae should result in a longer body and consequently a proportionally shorter head.

We checked for a possible segmental effect in recent *ASmg* and *SSmg* males and females separately, applying the method described below (exemplified with data from *SSmg* males). The vertebral column runs through the whole body and from radiographs it was estimated that about four vertebrae lie behind the operculum, that is, within the range of *Hl*.

*Sl* − *Hl* = *Bl* (Body length).

Mean *Sl* = 107.2 mm

Mean *Hl* = 25.4 mm

Mean *Vc* = 56.3 resulting in 52.3 within the region considered as *Bl*.

Consequently, Mean *Bl* = 107.2–25.4 mm = 81.8 mm

Mean vertebrae length for an average male: *SSmg* = 81.8/52.3 = 1.56 mm

Mean values where applied to draw the dashed lines in Figure A1 and elucidate hypothetical relationships between *Hl* and *Vc*, assuming a 100% segmental effect (when vertebrae are added or excluded from the average specimens). The graphic presentation (Figure A1) reveals a stronger tendency for a segmental effect in *SSmg* (close to significant in males) than in *ASmg*. Further, the difference in proportional *Hl* between *SS* and *AS* is influenced by, but far from fully explained by, a segmental effect. This is most easily seen when comparing *SS* and *AS* having 56–58 vertebrae, a range represented in both forms.

### 6. PCA FOR RECENT MATERIAL FROM LAKE FEGEN

Adjustments for size in a morphologically heterogeneous sample are often not fully doable applying a pooled among‐group regression as it will generate size‐dependent residuals (Bookstein et al., 1985). In a principal component analysis, PC I is often referred to as “size,” whereas PC II and further components are less correlated to size and therefore referred to as “shape” (Bookstein et al., 1985).

In the recent data set, growth of head in relation to standard length and growth of eye in relation to head length, respectively, appear isometric (Figures 7 and A6 below). In Figure A2a, there is strong correlation between PC I and size (*Sl*) as expected (see above), whereas Figure A2b reveals no obvious correlation between PC I and PC II, the former explaining about 86% of the total variation (Table A5). The selection of ripe and running specimens only, divided into spring‐ and autumn‐spawners, confirms size independence for PC II also for these subsets of recent specimens (Figure A2c).

**Table A5:** Character loadings on principal component I–II for three morphometric characters from the recent (1995–2008, *n* = 376) data set of *C. albula* from L. Fegen

	PC I	PC II
Standard length *Sl*	0.110	0.041
Head length *Hl*	0.107	0.005
Eye diameter *Ed*	0.094	−0.054
Percent of total variance explained	85.8	12.3

### 7. PCA FOR HISTORICAL MATERIAL FROM LAKE FEGEN

**Table A6:** Character loadings on principal components I–V for seven morphometric character from the historical data set of *C. albula* from Lake Fegen (Fegen *SSrr* and *ASrr* in Table A1)

	PC 1	PC II	PC III	PC IV	PC V
Total length *Tl*	0.046	0.017	0.016	0.015	0.005
Eye diameter *Ed*	−0.028	0.089	−0.023	0.027	0.010
Head length *Hl*	0.027	0.040	0.011	0.012	−0.028
Snout length	0.017	0.067	0.008	−0.049	0.002
Snout to dorsal fin	0.068	0.006	0.025	0.011	0.004
Snout to pelvic fin^a^	0.058	0.003	0.030	0.004	0.010
Body depth at Dorsal fin	0.098	−0.011	−0.054	−0.004	−0.001
Percent of total variance explained	45.2	30.5	11.5	7.73	2.22

a Included in original protocols but not in Svärdson (1979).

**Table A7:** Character loadings on principal components I–V for six meristic characters from the historical data set of *C. albula* from Lake Fegen (Fegen *SSrr* and *ASrr* in Table A1)

	PC 1	PC II	PC III	PC IV	PC V
Scales along lateral line	0.213	−0.964	0.027	−0.135	0.057
Dorsal fin rays	0.688	0.215	−0.029	−0.650	−0.134
Anal fin rays	0.676	0.113	0.204	0.059	0.695
Pectoral fin rays	0.394	−0.014	−0.908	0.102	0.073
Gill rakes	0.758	0.013	0.120	0.460	−0.240
Vertebrae *Vc*	0.803	−0.030	0.178	0.059	−0.295
Percent of total variance explained	39.2	16.5	15.2	11.2	10.9

### 9. ISOLATION WITH MIGRATION MODEL: PARAMETERS AND PRIORS

**Table A8:** Demographic parameters with prior distributions analyzed using popabc (under an Isolation with migration model)

Parameter	Description	Prior distribution
*N*_e1_	Effective population size (*AS*)	Uniform (10, 5,000)
N_e2_	Effective population size (*SS*)	Uniform (10, 5,000)
*N*_eA_	Effective population size (ancestral population)	Uniform (1,000, 20,000)
*m*_1_	Migration rate (gene flow) into *AS*	Uniform (0, 0.025)
*m*_2_	Migration rate (gene flow) into *SS*	Uniform (0, 0.025)
*t*	Divergence time (in generations)	Uniform (1, 2,000)

### 10. DIFFERENCE IN *VC* BETWEEN *SS* AND *AS* IN HISTORICAL AND RECENT MATERIAL

Irrespective of the different methods for counting vertebrae (historic vs. recent; see above), the absolute difference in average numbers between the two groups *AS* and *SS* in L. Fegen has decreased over time; from about 3.3 vertebrae in the 1950–1960s to about 1.5 in the 1990–2000s.

To test if the difference in *Vc* between *SS* and *AS* has changed significantly over time, the historic and contemporary data sets were randomly resampled 10,000 times. As shown by nonoverlapping 95% confidence intervals (Figure A5), the reduced difference in *Vc* between *SS* and *AS* over time is highly significant.

### 13. TEST FOR NON‐NEUTRAL LOCI

**Table A9:** Result from outlier analysis with lositan. Prob. is the proportion of simulated *F*_ST_‐estimates smaller than the observed one

Locus	*H*_E_	*F*_ST_	Prob.	1−Prob.
*BWF1*	0.90	0.16	.987	0.013
*BWF2*	0.54	0.01	.330	0.670
*Cisco126*	0.54	0.13	.811	0.189
*Cisco157*	0.48	0.09	.794	0.206
*Cisco90*	0.52	0.02	.424	0.576
*Cocl23*	0.30	0.02	.515	0.485
*Sfo23*	0.96	0.03	.777	0.223
*Sfo8*	0.43	0.00	.352	0.648
*Str73*	0.01	0.00	.500	0.500

### 14. GENETIC BOTTLENECKS

**Table A10:** Tests with bottleneck for deviations from expected heterozygosity at mutation‐drift equilibrium assuming two‐phase (TPM) and stepwise (SMM) models of mutation (Cornuet & Luikart, 1996)

Pop. assemblage	Sample	*n*	TPM					SMM			
			*H*_E_	*HE*	*HD*	Prob.	*H*_EQ_	*HE*	*HD*	Prob.	*H*_EQ_
1	Fegen *SSmg*	145	0.44	3	4	.77	0.46	3	4	.77	0.47
	Fegen *ASmg*	231	0.46	1	7	1.00	0.65	1	7	1.00	0.66
	Bolmen	53	0.53	1	7	.99	0.60	1	7	.99	0.61
	St Hålsjön	50	0.41	1	6	1.00	0.48	1	6	1.00	0.49
	Åsnen	40	0.47	1	6	.98	0.56	1	6	.98	0.57
	Åsunden	29	0.56	0	8	1.00	0.66	0	8	1.00	0.67
	Ören	10	0.49	4	4	.58	0.54	4	4	.68	0.55
2	S Dellen	39	0.62	3	5	.97	0.70	2	6	.99	0.71
	N Dellen	50	0.65	1	7	.99	0.73	0	8	1.00	0.74
	Kalix	32	0.65	2	6	.98	0.75	2	6	.99	0.76
	E Mälaren	55	0.63	4	4	.81	0.66	4	4	.88	0.67
	W Mälaren	55	0.64	5	3	.68	0.65	4	4	.81	0.66
	Rössjön	25	0.40	2	4	.92	0.41	2	4	.95	0.42
	Siljan	31	0.67	3	5	.88	0.73	3	5	.98	0.74
	Vänern	48	0.68	5	3	.63	0.71	5	3	.68	0.72
	Vättern	32	0.66	3	5	.97	0.72	2	6	.99	0.73

Note

*H*_E_ and *H*_EQ_ are the observed and expected heterozygosity, respectively, whereas *HE* and *HD* are the number of polymorphic loci showing excess and deficiency of heterozygotes. Prob. (probability) from one‐tailed Wilcoxon tests for heterozygote excess.
